# Ultra-long air-stability of n-type carbon nanotube films with low thermal conductivity and all-carbon thermoelectric generators

**DOI:** 10.1038/s41598-022-26108-y

**Published:** 2022-12-14

**Authors:** Yuki Amma, Katsuma Miura, Sho Nagata, Tsuyoshi Nishi, Shugo Miyake, Koji Miyazaki, Masayuki Takashiri

**Affiliations:** 1 Department of Materials Science, Tokai University, 4-1-1 Kitakaname, Hiratsuka, Kanagawa 259-1292 Japan; 2 Graduate School of Science and Engineering, Ibaraki University, 2-1-1 Bunkyo, Hitachi, Ibaraki 316-8511 Japan; 3 Department of Mechanical Engineering, Kobe City College of Technology, 8-3 Gakuenhigashi-Machi, Nishi-Ku, Kobe, Hyogo 651-2194 Japan; 4 Department of Mechanical and Control Engineering, Kyushu Institute of Technology, 1-1 Sensui, Tobata-Ku, Kitakyushu, Fukuoka 804-8550 Japan

**Keywords:** Thermoelectrics, Carbon nanotubes and fullerenes

## Abstract

This report presents n-type single-walled carbon nanotubes (SWCNT) films with ultra-long air stability using a cationic surfactant and demonstrates that the n-type Seebeck coefficient can be maintained for more than two years, which is the highest stability reported thus far to the best of our knowledge. Furthermore, the SWCNT films exhibit an extremely low thermal conductivity of 0.62 ± 0.08 W/(m·K) in the in-plane direction, which is very useful for thin-film TEGs. We fabricated all-carbon-nanotube TEGs, which use p-type SWCNT films and the n-type SWCNT films developed, and their air-stability was investigated. The TEGs did not degrade for 160 days and exhibited an output voltage of 24 mV, with a maximum power of 0.4 µW at a temperature difference of 60 K. These results open a pathway to enable the widespread use of carbon nanotube TEGs as power sources in IoT sensors.

## Introduction

Carbon nanotubes (CNTs) have several applications in fields such as electronics^1–3^, energy^4–6^, and functional materials^7–9^. Further, they have atomic structures in which hexagonal carbon is spirally arranged around the axis of a cylinder^10^. CNTs are generally classified as single-walled CNTs (SWCNTs) and multiwalled CNTs (MWCNTs) based on their structures. Compared with MWCNTs, SWCNTs have several superior properties, including extremely high electrical and thermal conductivities. Recently, the limitation of high SWCNT manufacturing costs has been overcome because of new synthesis methods^11,12^. In this context, it has become feasible to develop applications that require high-quality SWCNTs in large quantities.

SWCNTs are used in the development of thermoelectric generators (TEGs) that convert heat energy directly into electrical energy via the Seebeck effect^13–15^. TEGs based on SWCNTs exhibit flexibility, light weight, and moderately high thermoelectric properties near 300 K. Therefore, they can potentially be used as power supplies for Internet of Things (IoT) sensors^16–18^. Notably, it is often necessary to install several sensors with power supplies, including in narrow and bending regions, to enable the efficient use of IoT sensor networks. In general, TEGs consist of numerous n- and p-type thermoelectric elements that are alternately connected in series^19–21^. However, it is quite challenging to fabricate n-type SWCNTs with long-term stability in air. This difficulty exists because pristine SWCNTs exhibit n-type properties, which immediately change to p-type when oxygen molecules are adsorbed on the SWCNT surfaces; thus, electrons on the SWCNTs are transferred to the oxygen molecules^22–24^.

To overcome this limitation, several scholars have attempted and proposed methods to obtain n-type SWCNTs with long-term stability in air^25–30^. Nonoguchi et al. reported that salt-coordinated n-type SWCNTs exhibited excellent air stability for long durations, even at 100°C^25^. Hata et al. recently reported that polymer-sealed SWCNTs, including 1,2-diphenylhydrazine, were chemically stable for more than a month under accelerated aging conditions^26^. These pioneering studies motivated us to investigate air-stable n-type SWCNTs using facile processes. In our recent studies, n-type SWCNT films were prepared using different anionic surfactants, followed by heat treatment^31,32^. Among them, the SWCNT films with sodium dodecylbenzenesulfonate (SDBS) exhibited an n-type Seebeck coefficient of approximately − 50 µV/K for 14 days.

In this study, to extend the time for which the n-type Seebeck coefficient can be stably maintained, we used cationic surfactants dispersed in SWCNTs. The molecules in cationic surfactants are strongly attached to the CNT surfaces owing to cation-π orbital interactions, in contrast to those in anionic surfactants^25,33^. However, the dispersibility of cationic surfactants is lower than that of anionic surfactants. Therefore, we investigated several cationic surfactants dispersed in SWCNTs and estimated their thermoelectric properties with regard to air stability. The next step was to prepare all-carbon TEGs^34^. We prepared all-carbon TEGs, which consisted of p-type SWCNT films and SWCNT films with a cationic surfactant (n-type) on a flexible substrate, and measured the TEG performance.

## Results and discussion

**Figure 1:**
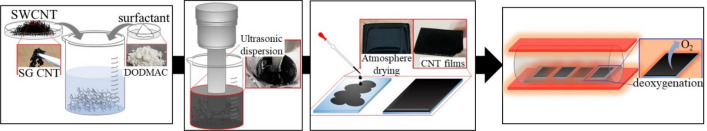
Fabrication process of SWCNT films with surfactants.

The fabrication of the SWCNT films using cationic surfactants was based on our recent work that demonstrated the fabrication of SWCNT films using anionic surfactants^31^. The SWCNT films (Fig. 1) were fabricated by drop-casting a dispersion solution of SWCNT powders and cationic surfactant onto a glass substrate, followed by heat treatment. We used two types of cationic surfactants: cetylpyridinium chloride (CPC) and dimethyl distearylammonium chloride (DODMAC). For reference, an anionic surfactant, SDBS, which exhibited the best performance among various anionic surfactants used in the preparation of SWCNT films in our previous study, was added to the SWCNT films^31^. Their molecular structures are shown in the Supplemental information (Figure S1).

**Figure 2:**
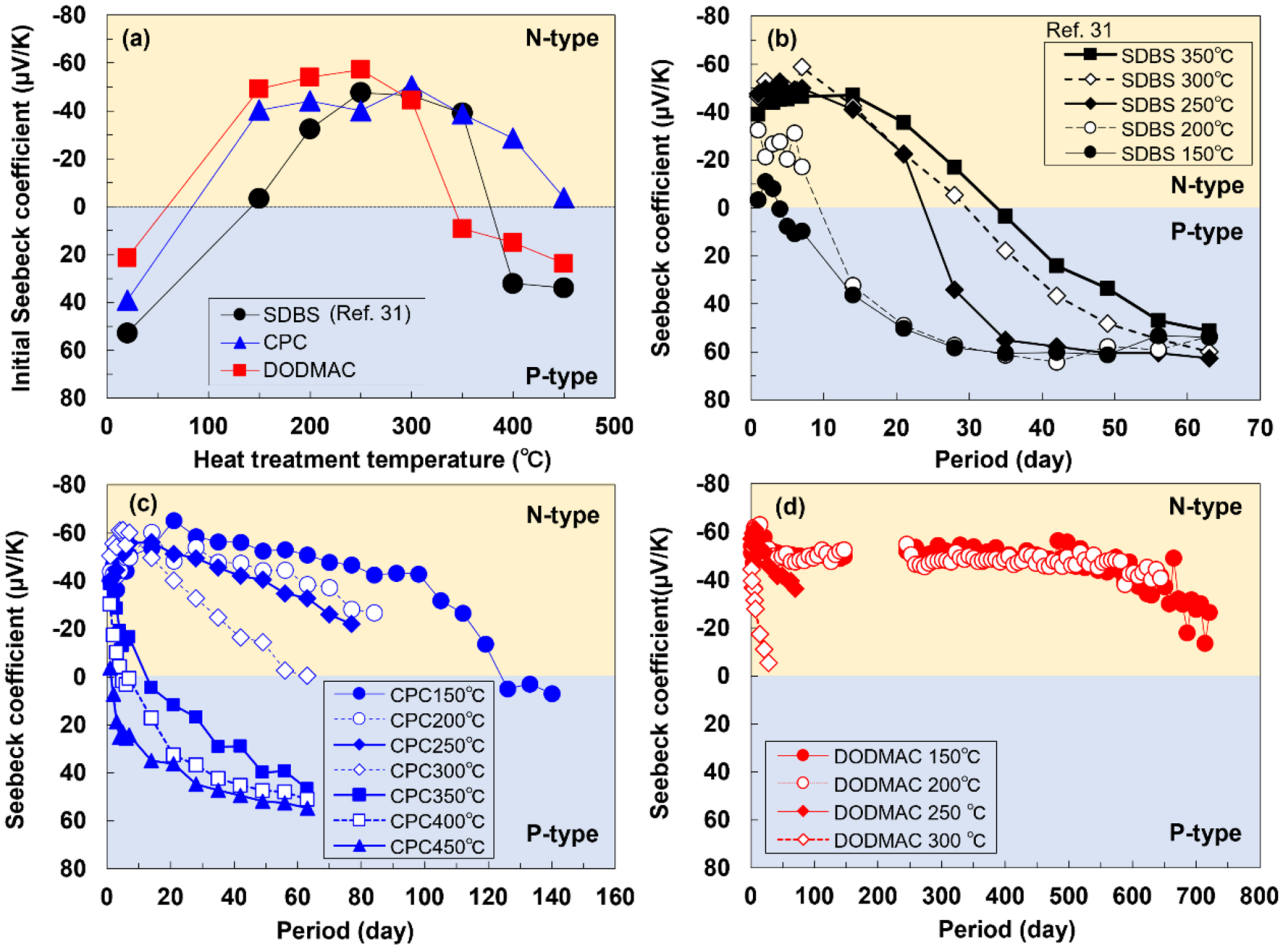
**(a)** Relationship between the initial Seebeck coefficient of different surfactants and heat treatment temperatures. Changes in Seebeck coefficients of SWCNT films over time with different surfactants and heat-treatment temperatures. SWCNT film with (**b)** SDBS, (**c)** CPC, and (**d)** DODMAC.

The Seebeck coefficient (initial value) as a function of heat treatment temperature of SWCNT films with different surfactants is shown in Fig. 2a. Without heat treatment (plotted at 20°C), each SWCNT film, regardless of the surfactant, exhibits positive Seebeck coefficients (p-type). At 150°C, the SWCNT films with cationic surfactants exhibit negative Seebeck coefficients ranging from − 40 to − 50 µV/K, and the SWCNT film with SDBS (anionic surfactant), the SDBS/SWCNT film, exhibits a near-zero Seebeck coefficient. The SWCNT film with DODMAC (cationic surfactant), the DODMAC/SWCNT film, exhibited the maximum negative value of the Seebeck coefficient (− 57 µV/K) at 250°C, and this negative value rapidly increases at temperatures from 300 to 350°C and becomes positive. The SWCNT films with CPC, CPC/SWCNT films, exhibited similar Seebeck coefficients of approximately − 40 µV/K in the temperature range of 150–350°C, and the absolute value of the Seebeck coefficient decreases at temperatures above 400°C. Upon using SDBS, the Seebeck coefficients become increasingly negative, and the high negative values stabilize at 250°C, which is 100°C higher than those of the SWCNT films prepared using cationic surfactants. Subsequently, the Seebeck coefficients become positive at temperatures above 400°C. The electrical conductivity as a function of heat treatment temperature of the SWCNT films with different surfactants is shown in the Supplemental information (Figure S2a). The changes in the Seebeck coefficients of the SWCNT films over time with different surfactants and heat treatment temperatures are shown in Fig. 2b–d. As shown in Fig. 2b, at 350°C, the SDBS/SWCNT films maintain a stable n-type Seebeck coefficient of approximately − 50 µV/K for 14 days, and the Seebeck coefficient changes to a positive value at 35 days^31^. As shown in Fig. 2c, the CPC/SWCNT films heated to a temperature lower than 350°C exhibit moderately stable n-type Seebeck coefficients. In particular, the highest stability is observed in the film heated to 150°C; the SWCNT films maintain stable n-type Seebeck coefficients of approximately − 50 µV/K for 98 days, and the Seebeck coefficients change to positive values after 120 days. As shown in Fig. 2d, the Seebeck coefficients of the DODMAC/SWCNT films exhibit significantly high air stability owing to the optimized heat treatment. When the heat treatment temperature is fixed at 150 and 200°C, n-type Seebeck coefficients of approximately − 50 µV/K are maintained for 665 days. After this period, the Seebeck coefficients gradually decrease; however, the n-type property is maintained for 721 days. To the best of our knowledge, this is the longest n-type air stability duration reported so far for n-type SWCNTs. Typical values of n-type air stability reported so far are shown in in the Supplemental information (Table S1)^26–31^. Notably, the properties of n-type SWCNTs depend on their diameter; 3–5 nm diameter SWCNTs were used here, which commonly exhibit excellent properties. The sample maintained at 200°C fractured after 644 days owing to repeated measurements. In addition, the interruption in the measurement period between 150 and 250 days was due to COVID-19 restrictions. Therefore, we demonstrated that the ultra-long air stability of n-type SWCNT films was achieved using a cationic surfactant, DODMAC, at a moderately low heat-treatment temperature. This low-temperature heat treatment contributes to decreasing the manufacturing costs by factors such as enabling the use of inexpensive substrates with low heat resistance and reducing the heater power consumption. The changes in the electrical conductivity of SWCNT films over time with different surfactants and heat-treatment temperatures are shown in the Supplemental information (Figures S2b–d).

**Figure 3:**
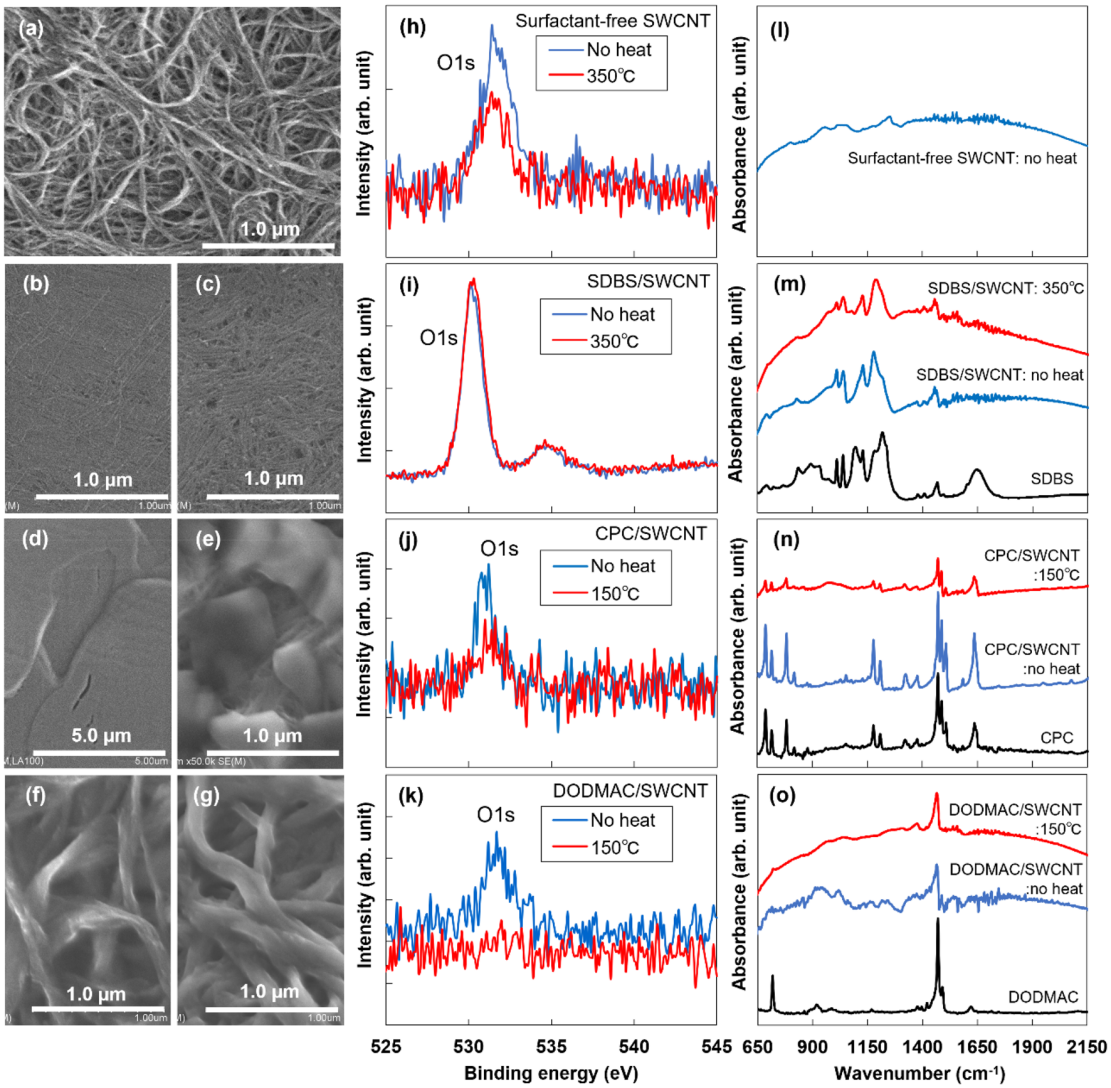
Surface morphologies of SWCNT films with different surfactants and heat-treatment temperatures observed by SEM. (**a)** Surfactant-free, no heat treatment. (**b)** SDBS, no heat treatment. **c** SDBS, 350°C. (**d)** CPC, no heat-treatment. (**e)** CPC, 150°C. (**f)** DODMAC, no heat treatment. (**g)** DODMAC, 150°C. Intensities of O1s spectra of SWCNT films with different surfactants and heat-treatment temperatures, observed using X-ray photoelectron spectroscopy (XPS). (**h)** Surfactant-free. (**i)** SDBS. (**j)** CPC. (**k)** DODMAC. Absorbance of SWCNT films with different surfactants and heat-treatment temperatures, observed using FTIR. (**l)** Surfactant-free. (**m)** SDBS. (**n)** CPC. (**o)** DODMAC.

Surface SEM images of the SWCNT films with different surfactants are shown in Fig. 3a–g. Based on the SEM images, no heat treatment and the heat treatment temperature that enabled the highest air stability were selected for each surfactant. The SEM images corresponding to the various heat treatment temperatures in this study are shown in the Supplemental information (Figure S3). A surface SEM image of the surfactant-free SWCNT film without heat treatment shows the stacking of numerous entangled SWCNT bundles with various diameters (Fig. 3a). In the SDBS/SWCNT film without heat treatment, SDBS molecules fill the gaps between the SWCNT bundles, and the film surface is flat (Fig. 3b). In the SDBS/SWCNT film heated to 350°C, the film surface is slightly rough because of the slight evaporation of SDBS surfactant (Fig. 3c). In the CPC/SWCNT film without heat treatment, the CPC surfactant completely covers the surface (Fig. 3d). In the CPC/SWCNT film heated to 150°C, random-shaped crystal-like structures are formed on the surface, and the underlying SWCNT surface can be partially observed (Fig. 3e). The change in surface morphology occurred because of the low melting point of CPC (77°C); the CPC melted during the heat treatment, then recrystallized and aggregated in the cooling process. In the DODMAC/SWCNT film without heat treatment, the SWCNT bundles are fully coated by the surfactant, and the film surface is moderately rough (Fig. 3f). In the DODMAC/SWCNT film heated to 150°C, the surface morphology is almost the same as that of the film without heat treatment (Fig. 3g). Notably, in the DODMAC/SWCNT film heated to 450°C, as shown in the Supplemental information (Figure S3), a similar morphology was observed even though the surfactant evaporated from the SWCNT surface, as demonstrated by FT-IR analysis in the Supplemental information (Figure S4). This finding indicates that the DODMAC surfactant thinly covers the SWCNT surface heated to 150°C. Therefore, the surface morphology of the SWCNT films is significantly affected by the type of surfactant used.

The existence of oxygen on the film surface was investigated by analyzing the O1s spectrum using XPS, as shown in Fig. 3h–k. The average depth of analysis for an XPS measurement is approximately 10 nm. The O1s spectrum of the surfactant-free film exhibits low intensity after heat treatment, but the O1s spectrum is still detected (Fig. 3h). This result indicates that oxygen is desorbed from the SWCNT surface by the heat treatment, and oxygen was subsequently adsorbed after the film was exposed to air. As shown in Fig. 3i, because the SDBS/SWCNT films contain oxygen as SO_3_^−^ in the SDBS structure, the adsorption of oxygen cannot be evaluated. In Fig. 3j, oxygen was detected in the untreated CPC/SWCNT film because of remaining water from the drop-casting process. The intensity of the O1s spectra of the CPC/SWCNT films decreased after the heat treatment, but oxygen was still present. This indicates that oxygen molecules re-adsorbed onto the exposed SWCNT surface, as shown in Fig. 3e. In Fig. 3k, oxygen from remaining water molecules was also detected in the untreated DODMAC/SWCNT film. However, the peak was no longer observed after the heat treatment, indicating that the water in the gaps within the DODMAC/SWCNT film was evaporated and that complete coverage was achieved.

The bonds between the SWCNTs and surfactants were investigated using Fourier-transform infrared spectroscopy (FTIR), as shown in Fig. 3l–o. For all the surfactants, the FTIR peaks of the films with and without heat treatment are derived from those of the SWCNTs and surfactants. Even after heat treatment, new bonds are not formed between the surfactants and SWCNTs. Therefore, surfactants exist in the films in a state such as the adsorption state on the SWCNTs.

When a cationic surfactant is used as the surfactant, the main body portion (cationic molecule) of the cationic surfactant is adsorbed well by the reduced SWCNTs (heated SWCNTs). The surfactant wets the SWCNT surfaces; subsequently, the SWCNT bundles are fully coated with DODMAC even though oxygen molecules remain near the SWCNT surfaces. Since the electron transfer rate from DODMAC to the SWCNTs exceeds that from the SWCNTs to the oxygen molecules, the DODMAC/SWCNT film exhibits an n-type Seebeck coefficient. To gather data on the n-type electron transfer reaction, we performed Raman spectroscopy analysis, the results of which are provided in the Supplemental information (Figure S5). Similar phenomena have been observed previously in alkali/crown-treated SWCNTs^25^. Therefore, DODMAC/SWCNT film exhibited and maintained n-type Seebeck coefficient for more than two years. The air stability of the CPC/SWCNT film is less than that of the DODMAC/SWCNT film because of the re-adsorption of oxygen by the separation of the surfactant layer and the SWCNT film.

Table 1 shows the thermal transport properties of the SWCNT film that exhibited the best air stability (DODMAC/SWCNT film heated at 150°C) after 250 days. For comparison, the properties of two types of SWCNT films are included in this table: a p-type SWCNT film without a surfactant, the surfactant-free/SWCNT film, which was prepared using the same SWCNTs and a similar fabrication process as that in this study^18^; an n-type SWCNT film with KOH and 18-crown-6-ether in dimethylformamide, a KOH_18-crown/SWCNT film, which shows one of the best results among n-type SWCNT films^25^. The in-plane and cross-plane thermal conductivities of the DODMAC/SWCNT film are 0.62 ± 0.08 and 0.40 ± 0.05 W/(m·K), respectively. Notably, the in-plane thermal conductivity is significantly lower than that of surfactant-free (5.4 ± 0.5 W/(m·K)) and KOH_18-crown/SWCNT films (39 ± 12 W/(m·K)). This trend is a very suitable characteristic for thin-film TEGs because the temperature difference can be increased in the in-plane direction. The ratio of in-plane and cross-plane thermal conductivities, i.e., the anisotropy in the thermal conductivity, of the DODMAC/SWCNT film (0.62/0.40) is lower than that of the surfactant-free/SWCNT film (5.5/0.16). A possible explanation that the DODMAC/SWCNT film exhibits low in-plane thermal conductivity and low anisotropy is provided in the Supplemental information (Figure S6). The in-plane power factor of the DODMAC/SWCNT film is 3.6 µW/(m·K^2^), which is lower than that of surfactant-free/SWCNT (26.7 µW/(m·K^2^) and KOH_18-crown/SWCNT films (2.05 × 10^2^ µW/(m·K^2^) because of the low electrical conductivity. However, the dimensionless figure of merit, *ZT*, is 1.7 × 10^–3^, which is comparable to those of surfactant-free/SWCNT (1.5 × 10^–3^) and KOH_18-crown/SWCNT films (2 × 10^–3^), because the DODMAC/SWCNT film has very low thermal conductivity in the in-plane direction. Therefore, we demonstrated that the DODMAC/SWCNT film with optimized heat treatment led to ultra-long air stability of the n-type property as well as a relatively high *ZT*.

**Table 1:** Thermal transport properties of SWCNT films.

Sample	Direction	*κ* [W/(m K)]	*S* [µV/K]	*σ* [S/cm]	*PF* [µW/(m K^2^)]	*ZT*	Remarks
N-type DODMAC film treated at 150°C after 250 days	In-plane	0.62 ± 0.08	− 55	12	3.6	1.7 × 10^–3^	This work meas. at 300 K
	Cross-plane	0.40 ± 0.05					
p-type SWCNT film without surfactant (no degradation)	In-plane	5.4 ± 0.5	55	88	26.7	1.5 × 10^–3^	Ref. 18 meas. at 300 K
	Cross-plane	0.06 ± 0.02					
N-type SWCNT film with KOH and 18-crown-6-ether in dimethylformamide	In-plane	39 ± 12	− 33	2.05 × 10^3^	2.3 × 10^2^	2 × 10^–3^	
	Cross-plane	0.02 ± 0.001	− 63	69	27	7 × 10^–2^	Ref. 25 meas. at 310 K

**Figure 4:**
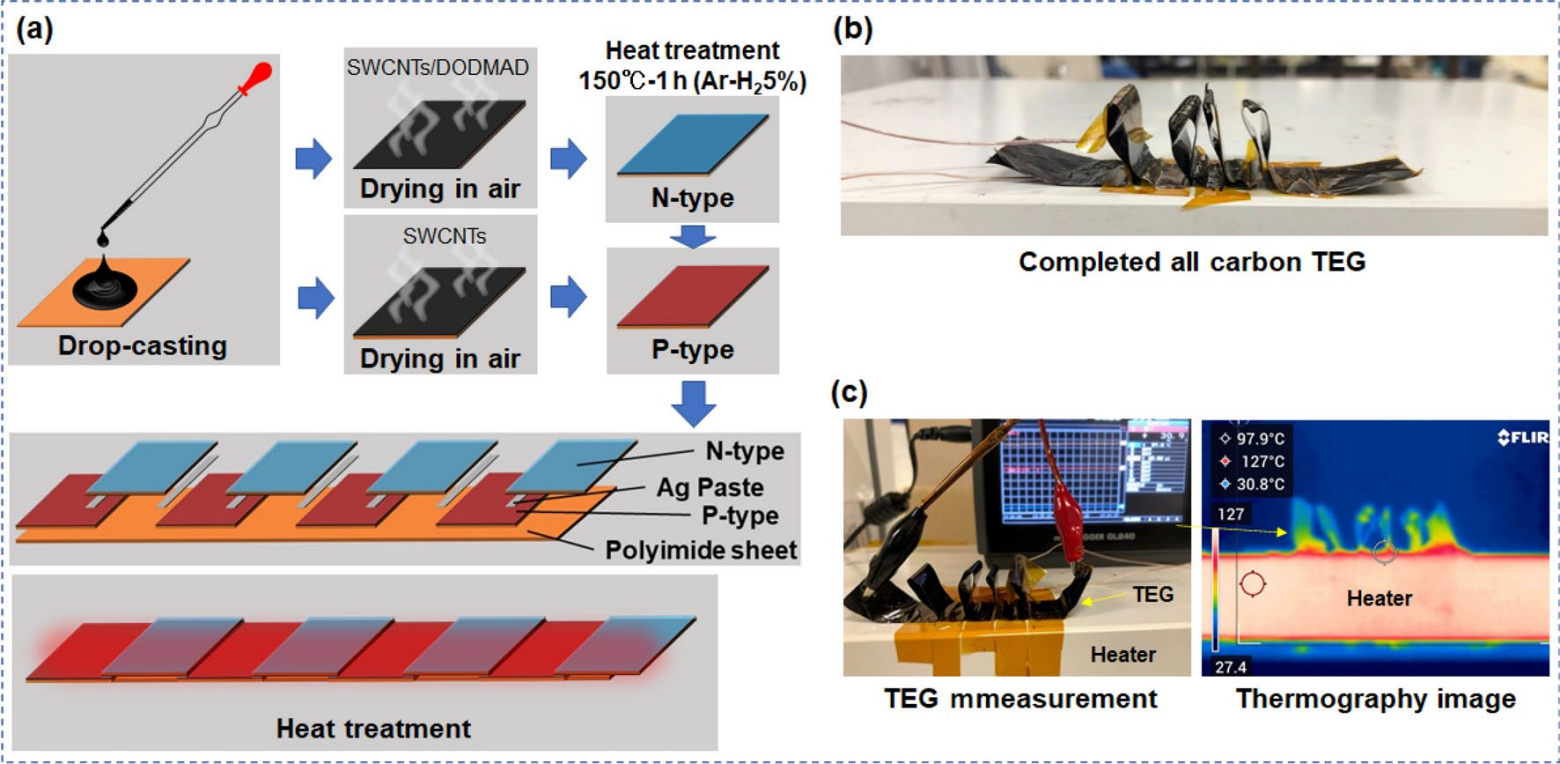
(**a)** Fabrication process of all-carbon TEG. (**b)** Photograph of completed all-carbon TEG. (**c)** Photograph of performance measurement of all-carbon TEG.

**Figure 5:**
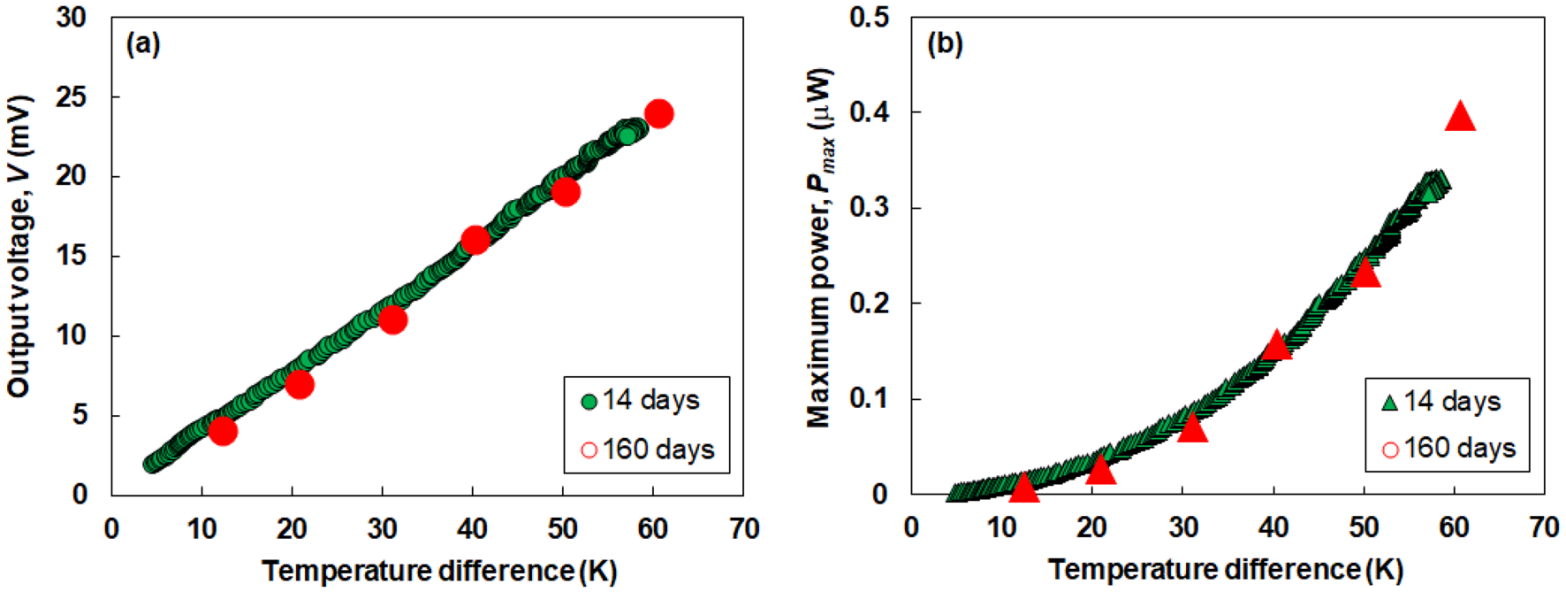
Performance of all-carbon TEG measured at 14th and 160th days after the TEG fabrication. (**a)** Output voltage and (**b)** maximum power.

All-carbon TEGs were fabricated on a flexible polyimide sheet by drop-casting, followed by heat treatment (Fig. 4a). The n-type SWCNT films were used as water-based dispersion solutions of SWCNT powders and DODMAC surfactants because of their ultra-long air stability. An ethanol-based dispersion solution of SWCNT powders with no surfactant was used to prepare the p-type SWCNT films. The TEG consisted of four pairs of n- and p-type SWCNT films, each of which was connected in series with a silver paste. To create a temperature difference in the TEG, it was bent so that the silver paste alternated between the top and bottom (Fig. 4b). The TEG was placed on a heater, and the output voltage *V* was measured while the temperature of the heater was controlled (Fig. 4c). Figure 5 shows the performance of the all-carbon TEG as a function of the applied temperature difference. To investigate the air stability of the all-carbon TEG, measurements are performed on the 14th and 160th days after the TEG is fabricated. As shown in Fig. 5a, the output voltage linearly increases as the temperature difference increased. The relationship between the temperature difference and the output voltage of the TEG measured on the 160th day is almost the same as that measured on the 14th day. The TEG measured on the 160th day exhibits an output voltage of 24 mV at a temperature difference of 60 K. As shown in Fig. 5b, the maximum power increases quadratically as the temperature difference is increased. The relationship between the temperature difference and the maximum power in the TEG measured on the 160th day is almost the same as that measured on the 14th day. The TEG measured on the 160th day exhibits the maximum power of 0.4 µW at a temperature difference of 60 K. Therefore, we demonstrate that all-carbon TEGs produced output voltage and electric power while the air stability was maintained for a long period. We achieved the target voltage for working IoT sensors (20 mV) but at a relatively large temperature difference. The subsequent challenge is to enhance the TEG performance at a small temperature difference by optimizing the TEG design.

## Conclusion

In summary, n-type SWCNT films exhibiting n-type Seebeck coefficients with prolonged air stability were achieved using cationic surfactants via a facile fabrication process. The cationic surfactants were mixed with SWCNTs, and the films were fabricated by drop casting followed by heat treatment. When DODMAC was used as the cationic surfactant and the heat-treatment temperature was set at 150 and 200°C, the Seebeck coefficient exhibited and maintained n-type properties for more than two years. A reason for this phenomenon is that DODMAC fully coated the SWCNTs even though oxygen molecules remained near the SWCNT surfaces; the preferential electron transfer between DODMAC and the SWCNTs therefore produced an n-type Seebeck coefficient in the resulting film. Furthermore, the SWCNT films exhibit extremely low thermal conductivity in the in-plane direction, which is very useful for thin-film TEGs. All-carbon TEGs (n-type: SWCNT/DODMAC and p-type: SWCNTs) were fabricated on a flexible polyimide sheet by drop-casting, followed by heat treatment. The TEGs do not degrade for 160 days and exhibit an output voltage of 24 mV and a maximum power of 0.4 µW at a temperature difference of 60 K. Although the performance remains insufficient, the results open a pathway to enable the widespread use of CNT TEGs as power sources for IoT sensors.

## Methods

### Materials

SWCNTs, known as super growth-CNTs (ZEONANO SG101), were supplied by Zeon Corporation. Cationic surfactants, CPC (Tokyo Chemical Industry Co.) and DODMAC (FUJIFILM Wako Pure Chemical), were used as received. For reference, the anionic surfactant, SDBS (Tokyo Chemical Industry Co.), was used as received.

### Preparation of SWCNT films with surfactants

SWCNTs with surfactants were ultrasonically dispersed in deionized water. We used two types of cationic surfactants, CPC and DODMAC, and an anionic surfactant, SDBS. The concentrations of the SWCNTs and surfactants in the deionized water were 0.2 and 1.0 wt%, respectively. An ultrasonic homogenizer (SONICS 85, AZONE Co.) was used to disperse the SWCNT powder completely. The SWCNT films were prepared on a glass substrate with a limited deposition area (2.5 × 2.0 cm) by drop-casting. The film thickness was approximately 10 µm. A pipette was used to drop-cast 0.9 ml of the SWCNT dispersion liquid onto the substrate. After drop-casting, the dispersion liquid was naturally dried under atmospheric conditions for approximately 24 h. The SWCNT films deposited on the glass substrates were heated in an electric furnace. The detailed procedures for heat treatment are available in our previous reports^35,36^. In brief, the furnace was filled with a mixture of argon (95%) and hydrogen (5%) gases at atmospheric pressure. Hydrogen gas was added to reduce the amount of oxygen atoms on the SWCNT film surface. The heat-treatment temperatures were set at 150, 200, 250, 300, 350, 400, and 450°C, and the treatment duration was 1 h. The sample was removed when the temperature in the furnace was less than 100°C.

### Measurement of thermoelectric properties

The in-plane Seebeck coefficient of the SWCNT films was measured near 300 K with an accuracy of ± 10%^37^. One end of the film was connected to a heat sink, and the other end was connected to a heater. The Seebeck coefficient was determined as the ratio of the potential difference along the membrane to the temperature difference measured using two 0.1-mm-diameter K-type thermocouples pressed against the membrane. The in-plane electrical conductivity of the SWCNT films was measured at a temperature near 300 K using a four-point probe method (Napson, RT-70 V) with an accuracy of ± 10%. To measure the time dependence of the Seebeck coefficient and electrical conductivity of the SWCNT films, the measurement was first performed at intervals of 1 day for a total of 7 days; thereafter, measurements were performed at intervals of 7 days for a total of 721 days. The in-plane and cross-plane thermal diffusivities, *D*_*in*_ and *D*_*cross*_, respectively, were measured by non-contact laser spot periodic heating radiation thermometry (Bethel Co., Thermowave analyzer) with an accuracy of ± 3%^38^. The in- and cross-plane thermal diffusivities, *D*_*in*_ and *D*_*cross*_, are 1.66 and 1.07 mm^2^/s, respectively. The specific heat was measured using a differential scanning calorimeter (Shimadzu, DSC-60) with an accuracy of ± 10%, and the value was 0.89 J/(g·K). The thermal conductivity can be determined from the thermal diffusivity (*D*), density (*ρ*), and specific heat (*C*_*p*_) based on the equation *κ* = *DρC*_*p*_. The density of the SWCNT film was measured as 0.42 g/cm^3^.

### Characterizations

The surface morphologies of the SWCNT films were analyzed using field emission scanning electron microscopy (FE-SEM; Hitachi S-4800) at an accelerating voltage of 3 kV. The chemical structures of the SWCNT films were characterized by XPS (ULVAC-PHI Quantum 2000) using Al Kα irradiation and FTIR (JASCO FT/IR-4200).

### Preparation of all-carbon TEGs

The fabrication process and conditions for the n-type DODMAC/SWCNT films were the same as those described in the section “Preparation of SWCNT films with surfactants,” except for the substrate used; a polyimide sheet (25 × 20 mm^2^, 125 µm thick) was used as the substrate for TEG fabrication. For TEG fabrication, the cationic surfactant DODMAC was used at a heat treatment temperature of 150°C because ultra-long air stability was achieved in the SWCNT films using this surfactant. After the heat treatment, the ends of the n- and p-type films were connected in series using a silver paste. The resulting TEG consisted of four pairs of n- and p-type SWCNT films. The prepared TEG was 230 mm long and 22 mm wide. To create a temperature difference in the TEG, it was bent so that the positions of the silver paste were at the top. The height of the bent TEG was 16 mm, and the shrunk size was 46 mm long and 22 mm wide.

### Performance measurements of all-carbon TEGs

The performance of the TEG was experimentally measured while the heater temperature was varied. The TEG was firmly attached to the heater, and the temperatures at the top and bottom of the TEG were measured using two thermocouples (K-type). Two Cu-wire electrodes were connected to the outermost SWCNT films to measure the output voltage. The two thermocouples and two Cu wires were connected to a data logger (GL240-SD midi LOGGER, GRAPHTEC). Thus, the relationship between the temperature difference between the top and bottom positions of the TEG and the output voltage was measured. The maximum power, *P*_*max*_, was calculated from the output voltage *V* and the measured total resistance of the TEG, *R*_*total*_, as follows: *P*_*max*_ = *V*^2^/4*R*_*total*_. Since the *R*_*total*_ changes with the device temperature, the temperature dependence of the *R*_*total*_ was evaluated as shown in the Supplemental information (Figure S7).

## Supplementary Information

Supplementary Information.

## Data Availability

The authors declare that most data supporting the findings of this study are available within the paper and its supplementary information files. The rest of the data generated during and/or analyzed during the current study are available from the corresponding author upon reasonable request.
