# Supplementary material for: Ultra-long air-stability of n-type carbon nanotube films with low thermal conductivity and all-carbon thermoelectric generators
Source: Sci Rep. 2022 Dec 14;12:21603. doi: 10.1038/s41598-022-26108-y (PMC9748887; doi:10.1038/s41598-022-26108-y)
Supplement: Supplementary file 1 — Supplementary Information. [file 41598_2022_26108_MOESM1_ESM.docx]

**Ultra-long air-stability of n-type carbon nanotube films with low thermal conductivity and all-carbon thermoelectric generators**

*Yuki Amma^1^, Katsuma Miura^1^, Sho Nagata^2^, Tsuyoshi Nishi^2^, Shugo Miyake^3^, Koji Miyazaki^4^ and Masayuki Takashiri^1*^*

^1^Department of Materials Science, Tokai University, 4-1-1 Kitakaname, Hiratsuka, Kanagawa 259-1292, Japan.

^2^Graduate School of Science and Engineering, Ibaraki University, 2-1-1 Bunkyo, Hitachi, Ibaraki, 316-8511, Japan.

^3^Department of Mechanical Engineering, Kobe City College of Technology, 8-3 Gakuenhigashi-machi, Nishi-ku, Kobe, Hyogo 651-2194, Japan.

^4^Department of Mechanical and Control Engineering, Kyushu Institute of Technology, 1-1 Sensui, Tobata-ku, Kitakyushu, Fukuoka 804-8550, Japan.

*E-mail: takashiri@tokai-u.jp


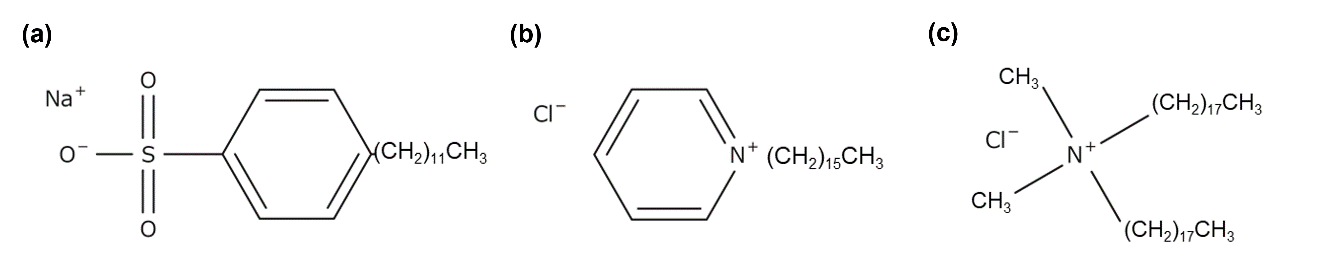
**Supplemental information**

**Figure S1.** Molecular structures of surfactants. a SDBS. b CPC. c DODMAC.

**Table S1.** Stability of n-type SWCNTs treated by various methods and materials

References

1. Y. Seki, K. Nagata, M. Takashiri, *Sci. Rep.* **2020**, *10*, 8104.
2. Y. Nakashima, *et al*. *ACS Appl. Nano Mater*. **2019**, *2*, 4703–4710.
3. Z. Wenbin, *et al.* *Nat. Commun.* **2017**, *8*, 14886.
4. S. Hata, *et al*. *Chem. Lett.* **2019**, *48*, 1109–1111.
5. Y. Nonoguchi, *et al.* *Adv. Funct. Mater.* **2016**, *26*, 3021–3028.
6. D. D. Freeman, K. Choi, C. Yu, *PLoS One* **2012**, *7*, e47822.
7. W. Guangbao, *et al.* *ACS Nano* **2017**, *11*, 5746–5752.

**
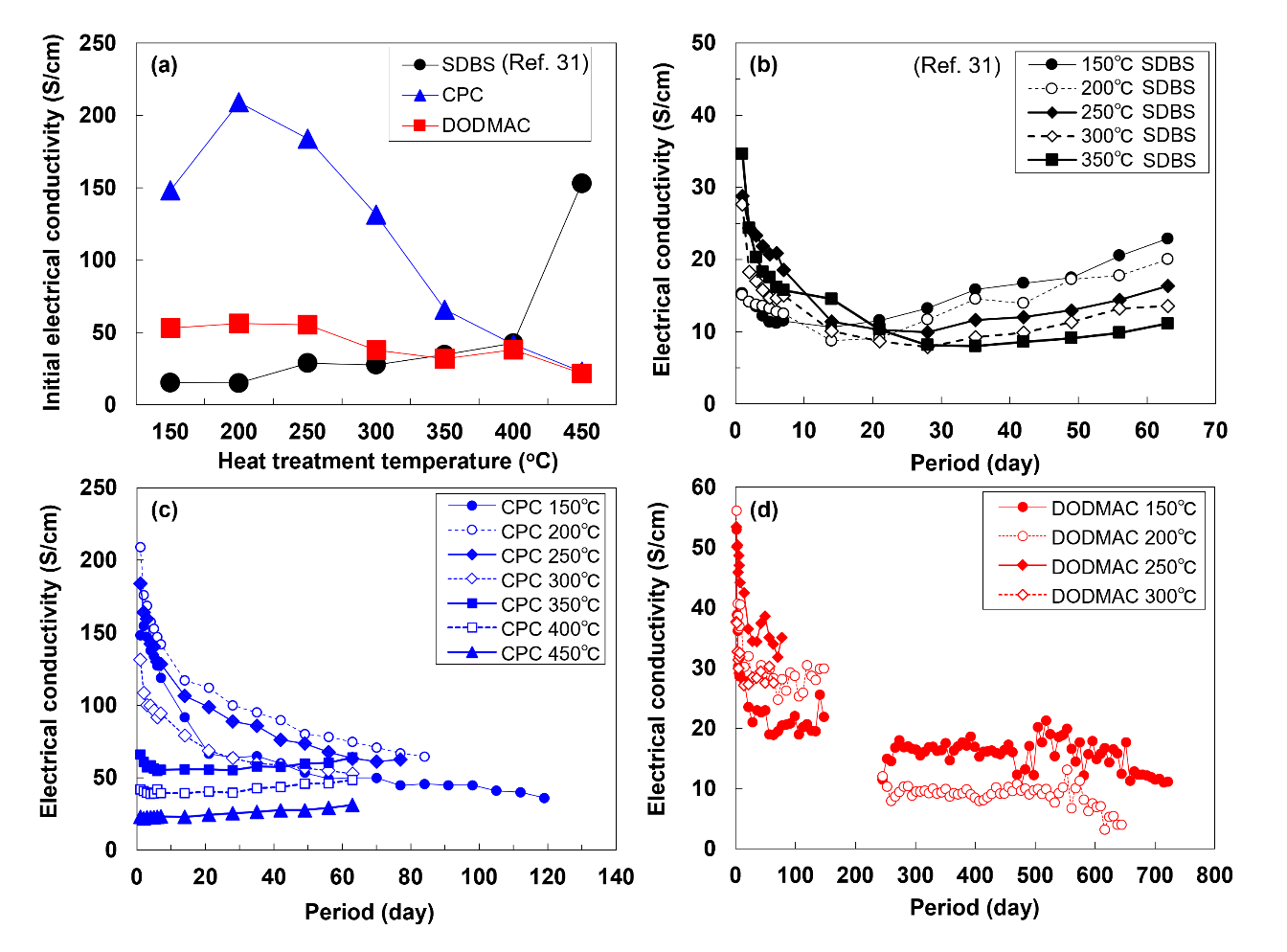
 Figure S2**: **a** Relationship between the initial electrical conductivity of different surfactant and heat treatment temperature. Chronological change in electrical conductivities of SWCNT films with different surfactants and heat-treatment temperatures. **b** SWCNT film with SDBS. **c** SWCNT film with CPC. **d** SWCNT film with DODMAC.

Notably, the electrical conductivity of the DODMAC/SWCNT film decreases over time, while the Seebeck coefficient remains relatively constant over the same period (**Figure 2**d). A clear mechanism for the difference has not yet been identified. One possibility is that the DODMAC/SWCNT films are damaged when their electrical conductivities are repeatedly measured using a four-probe method. In this method, four needles are pressed against the film, and small holes are opened in the films. When the measurement is repeated, the number of small holes increased, indicating accumulated damage. Thus, the number of current pathways is reduced after each measurement. As a result, the electrical conductivity decreases with time. Conversely, the Seebeck coefficient does not depend on the number of current pathways, thus it remains unchanged. A detailed study to clarify the mechanism of this conductivity reduction is currently underway by examining whether the damage to the films is reduced by expanding the measurement periods (once a month).


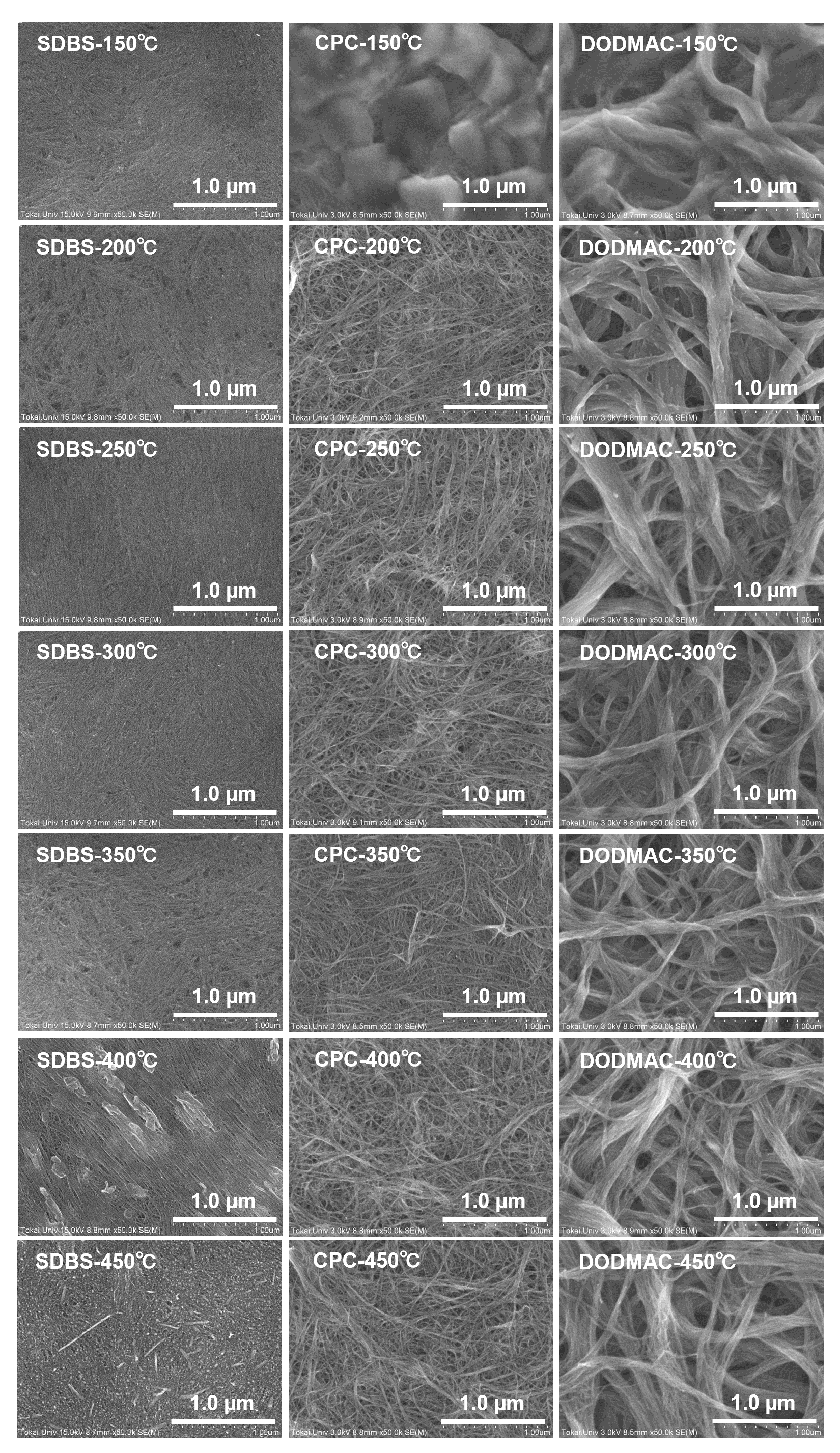


**Figure S3**: Surface morphologies of SWCNT films with different surfactants and heat-treatment temperatures, observed with SEM.

**
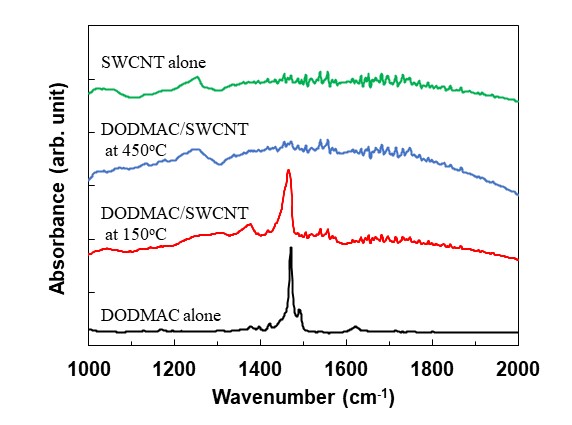
**

**Figure S4**. FT-IR spectra of SWCNT films with DODMAC at different heat-treatment temperatures, DODMAC alone, and SWCNT alone.

The FT-IR spectrum of DODMAC/SWCNT film at 150°C includes the signal from the DODMAC and SWCNT, indicating that the DODMAC covers the SWCNT surface. On the other hand, the FT-IR spectrum of DODMAC/SWCNT film at 450°C includes only the signal from the SWCNT, indicating that the DODMAC evaporates from the SWCNT surface.


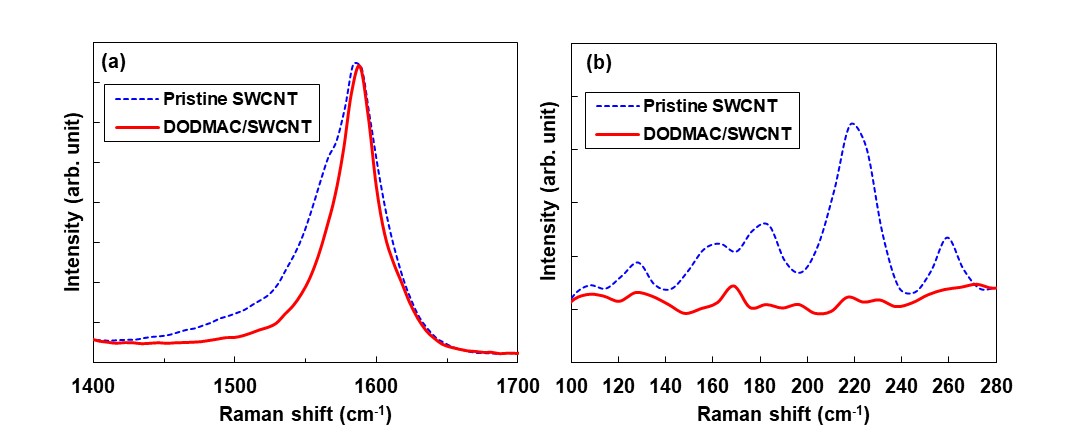


**Figure S5**. Normalized Raman spectra of Pristine SWCNT and DODMAC/SWCNT thermal treatment at 150^o^C in (a) G-band and (b) RBM regions.

To gather data on the electron transfer reaction in n-type doped film, we performed Raman spectroscopy. **Figure S5** shows the normalized Raman spectra (excitation wavelength = 785 nm) of pristine SWCNTs and the DODMAC/SWCNT film after a 150°C thermal treatment. In **Figure 5**a, there is no significant peak shift of the cooperative in-plane stretching mode of the aromatic rings (G-band) at 1589 cm^-1^ between the two samples. This may suggest that the n-type doping does not disturb the C-C bond strength. In **Figure 5**b, the intensity of the radial breathing (RMB) modes was effectively suppressed after doping with DODMAC. This result corroborates previous observations of the modulation of the sp^2^ carbon backbone with charge injection and/or ion adsorption (Takenobu et al., Nat. Mater. 2, 683 (2003)).


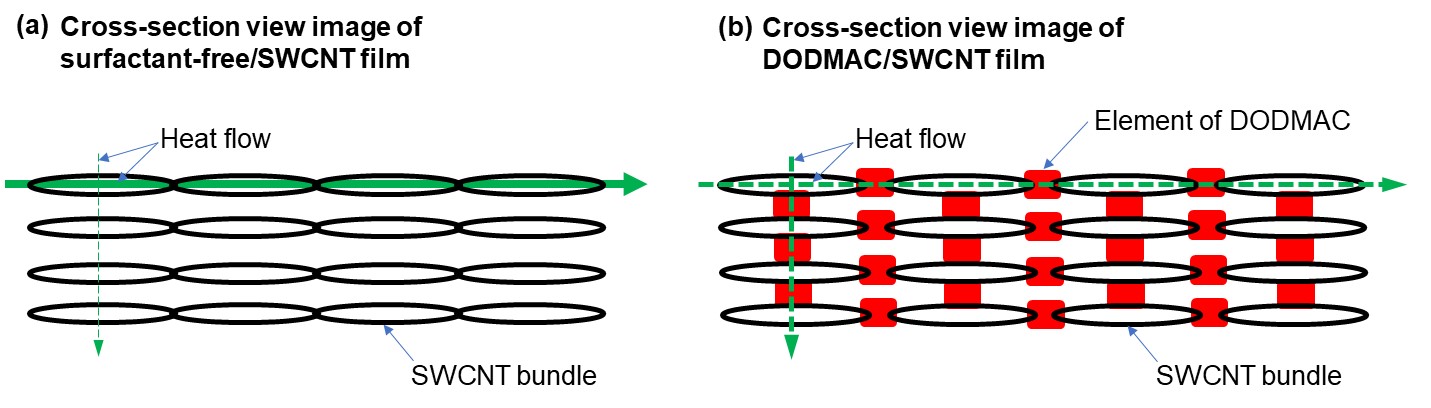


**Figure S6**. A model for explanation of the decrease in the in-plane thermal conductivity and low anisotropy occurring in DODMAC/SWCNT films by comparing that of the no-surfactant/SWCNT. Cross-section view images of (a) surfactant-free/SWCNT film and (b) DODMAC/SWCNT film.

Here, we discuss possible mechanisms explaining why the DODMOC/SWCNT film exhibits low in-plane thermal conductivity and low anisotropy in comparison to the surfactant-free SWCNT film. A possible reason why the SWCNTs (SG-CNTs) used in this study have a low thermal conductivity compared to other SWCNTs such as e-DIPS (Chiba et al., Sci. Rep. 11, 14707 (2021), Nonoguchi et al., Adv. Funct. Mater. 26, 3021–3028 (2016)) is because SG-CNTs have a higher defect density (Chiba et al., AIP Adv. 11, 015332 (2021)). Additional potential mechanisms are presented in **Figure S6**. **Figure S6**a shows a cross-sectional diagram of the surfactant-free SWCNT film. When a surfactant is not used to form the SWCNT film, the SWCNT bundles are strongly connected in the in-plane direction, but weakly in the cross-plane direction. As a result, in-plane thermal conductivity is relatively high, but lower in the cross-plane direction, thus leading to high anisotropy. **Figure S6**b shows a cross-sectional diagram of the DODMAC/SWCNT film. DODMAC is inserted between the SWCNT bundles in both directions. In the in-plane direction, the thermal conductivity decreases as the thermal conductivity of DODMAC is lower than that of the SWCNTs, thus heat transfer is effectively reduced between the neighboring SWCNTs. This would also explain why the in-plane electrical conductivity of the DODMAC/SWCNT film is lower than that of the surfactant-free SWCNT film. Conversely, the thermal conductivity in the cross-plane direction increases because the inserted DODMAC blocks occupy the gaps between the SWCNT bundles, leading to a decrease in anisotropy. A similar phenomenon has also been observed in a previous report using CNT composites where biomolecules were inserted at the nanotube junctions (Ito et al., Appl. Phys. Express 7, 065102 (2014).

**Figure S7**: Resistance of TEG applying temperature difference.
